# ChatGPT’s quiz skills in different otolaryngology subspecialties: an analysis of 2576 single-choice and multiple-choice board certification preparation questions

**DOI:** 10.1007/s00405-023-08051-4

**Published:** 2023-06-07

**Authors:** Cosima C. Hoch, Barbara Wollenberg, Jan-Christoffer Lüers, Samuel Knoedler, Leonard Knoedler, Konstantin Frank, Sebastian Cotofana, Michael Alfertshofer

**Affiliations:** 1 Department of Otolaryngology, Head and Neck Surgery, School of Medicine, Technical University of Munich (TUM), Ismaningerstrasse 22, 81675 Munich, Germany; 2 Department of Otorhinolaryngology, Head and Neck Surgery, Medical Faculty, University of Cologne, 50937 Cologne, Germany; 3 Division of Plastic Surgery, Brigham and Women’s Hospital, Harvard Medical School, Boston, MA 02152 USA; 4 Department of Plastic Surgery and Hand Surgery, Klinikum Rechts Der Isar, Technical University of Munich, Munich, Germany; 5 Division of Plastic and Reconstructive Surgery, Massachusetts General Hospital, Harvard Medical School, Boston, MA 02115 USA; 6 Ocean Clinic, Marbella, Spain; 7 Department of Dermatology, Erasmus Hospital, Rotterdam, The Netherlands; 8 Centre for Cutaneous Research, Blizard Institute, Queen Mary University of London, London, UK; 9 Division of Hand, Plastic and Aesthetic Surgery, Ludwig-Maximilians-University Munich, Munich, Germany

**Keywords:** ChatGPT, Artificial intelligence, AI, Otolaryngology quiz, Multiple-choice, Single-choice

## Abstract

**Purpose:**

With the increasing adoption of artificial intelligence (AI) in various domains, including healthcare, there is growing acceptance and interest in consulting AI models to provide medical information and advice. This study aimed to evaluate the accuracy of ChatGPT’s responses to practice quiz questions designed for otolaryngology board certification and decipher potential performance disparities across different otolaryngology subspecialties.

**Methods:**

A dataset covering 15 otolaryngology subspecialties was collected from an online learning platform funded by the German Society of Oto-Rhino-Laryngology, Head and Neck Surgery, designed for board certification examination preparation. These questions were entered into ChatGPT, with its responses being analyzed for accuracy and variance in performance.

**Results:**

The dataset included 2576 questions (479 multiple-choice and 2097 single-choice), of which 57% (*n* = 1475) were answered correctly by ChatGPT. An in-depth analysis of question style revealed that single-choice questions were associated with a significantly higher rate (*p* < 0.001) of correct responses (*n* = 1313; 63%) compared to multiple-choice questions (*n* = 162; 34%). Stratified by question categories, ChatGPT yielded the highest rate of correct responses (*n* = 151; 72%) in the field of allergology, whereas 7 out of 10 questions (*n* = 65; 71%) on legal otolaryngology aspects were answered incorrectly.

**Conclusion:**

The study reveals ChatGPT’s potential as a supplementary tool for otolaryngology board certification preparation. However, its propensity for errors in certain otolaryngology areas calls for further refinement. Future research should address these limitations to improve ChatGPT’s educational use. An approach, with expert collaboration, is recommended for the reliable and accurate integration of such AI models.

## Introduction

Artificial intelligence (AI) refers to the technology that aims to develop algorithms and computer systems capable of performing tasks that typically require human intelligence [1]. Therefore, the remit of AI is multi-faceted, reaching from language understanding through image and pattern recognition to decision making and problem solving [2, 3]. AI is based on machine learning, whereby computers are generally taught to learn from data, and deep learning, which leverages neural networks to facilitate pattern recognition and decision-making [4]. Specifically in the field of otolaryngology, the clinical applicability of AI is well-documented and includes the automation of classification tasks, analysis of clinical patient data, and simulation of preoperative surgical outcomes [1, 5–8].

Recently, ChatGPT, an interactive chatbot, has emerged as a revolutionary language-based AI model. Powered by the state-of-the-art GPT-4 language model and advanced deep learning techniques, ChatGPT is able to generate human-like responses across a broad spectrum of topics, covering both medical and non-medical domains.

As the popularity of ChatGPT continues to grow, an increasing number of users turn to this AI model for medical advice. Albeit previous studies have reported on ChatGPT’s ability to provide medical information [9–11], announcing a potential paradigm shift in medical education and clinical decision-making, a comprehensive and holistic investigation of ChatGPT’s performance in medical assessments remains to be conducted. As a result, there exists a knowledge gap regarding the utilization of ChatGPT for other board-style practice examinations, such as the German otolaryngology board examination. In addition, the performance of ChatGPT in subject-specific and subspecialty contexts has yet to be determined.

This study aims to evaluate the accuracy of ChatGPT’s responses to practice questions for the German otolaryngology board certification and delineate differences in performance across distinct subspecialties within this medical discipline. Our findings may contribute to the broader puzzle of understanding and utilizing AI and ChatGPT to advance medical education and improve clinical decision-making.

## Methods

### Question database

We used the question database of an online learning platform (https://hno.keelearning.de/), which offers quiz-style questions to prepare for the German otolaryngology board certification. The platform is funded by the German Society of Oto-Rhino-Laryngology, Head and Neck Surgery, and encompasses a comprehensive range of 15 distinct otolaryngology subspecialties. These subspecialties include allergology, audiology, ENT tumors, face and neck, inner ear and skull base, larynx, middle ear, oral cavity and pharynx, nose and sinuses, phoniatrics, salivary glands, sleep medicine, vestibular system, and legal aspects. To ensure the validity of the study, any image-based questions were excluded from the analysis. A total of 2576 questions were included and categorized by question style into multiple-choice (479 questions) and single-choice (2097 questions). Prior to the start of the study, official permission to use the questions for research purposes was obtained from the copyright holder.

### ChatGPT prompts and analysis

The testing of the AI model was conducted by C.C.H. and M.A. between May 5th and May 7th, 2023, by manually inputting the questions into the most recent version of ChatGPT (May 3rd version) on the respective website (https://chat.openai.com). It is important to note that the questions were entered into the AI system only once during the testing process. To account for variations in question formats, two distinct prompts were employed when asking ChatGPT to respond to quiz-style questions with four options.

For single-choice style questions, the following prompt was used:

(A) “Please answer the following question. Note that only one option is correct:

Single-choice question

- *Option A*
- *Option B*
- *Option C*
- *Option D*”

For questions in the multiple-choice format, we included the following prompt:

(B) “Please answer the following question. Note that several options may be correct:

Multiple-choice question

- *Option A*
- *Option B*
- *Option C*
- *Option D*”

**Fig. 1:**
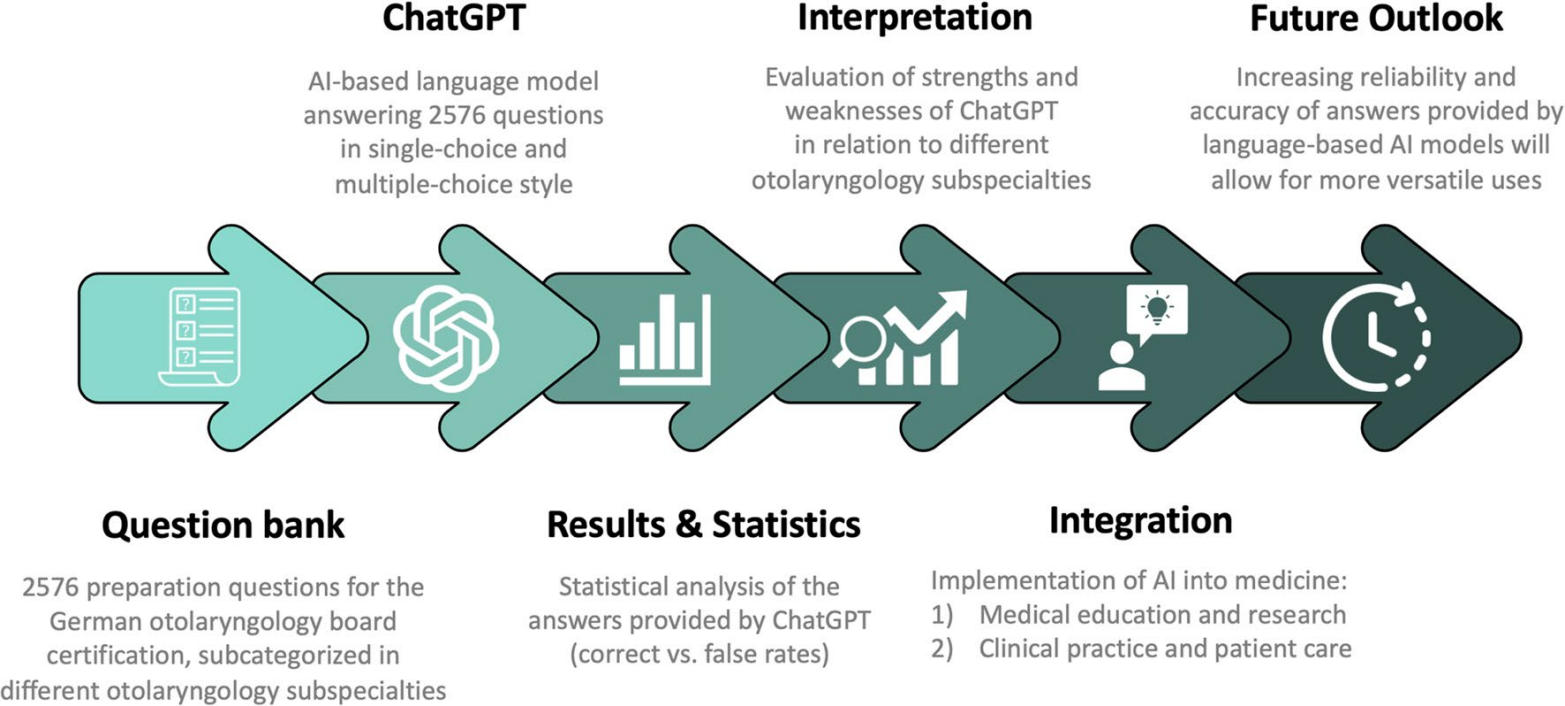
Workflow summarizing the methodology used in the study, as well as showing the integration of intensified research on artificial intelligence in medicine

**Fig. 2:**
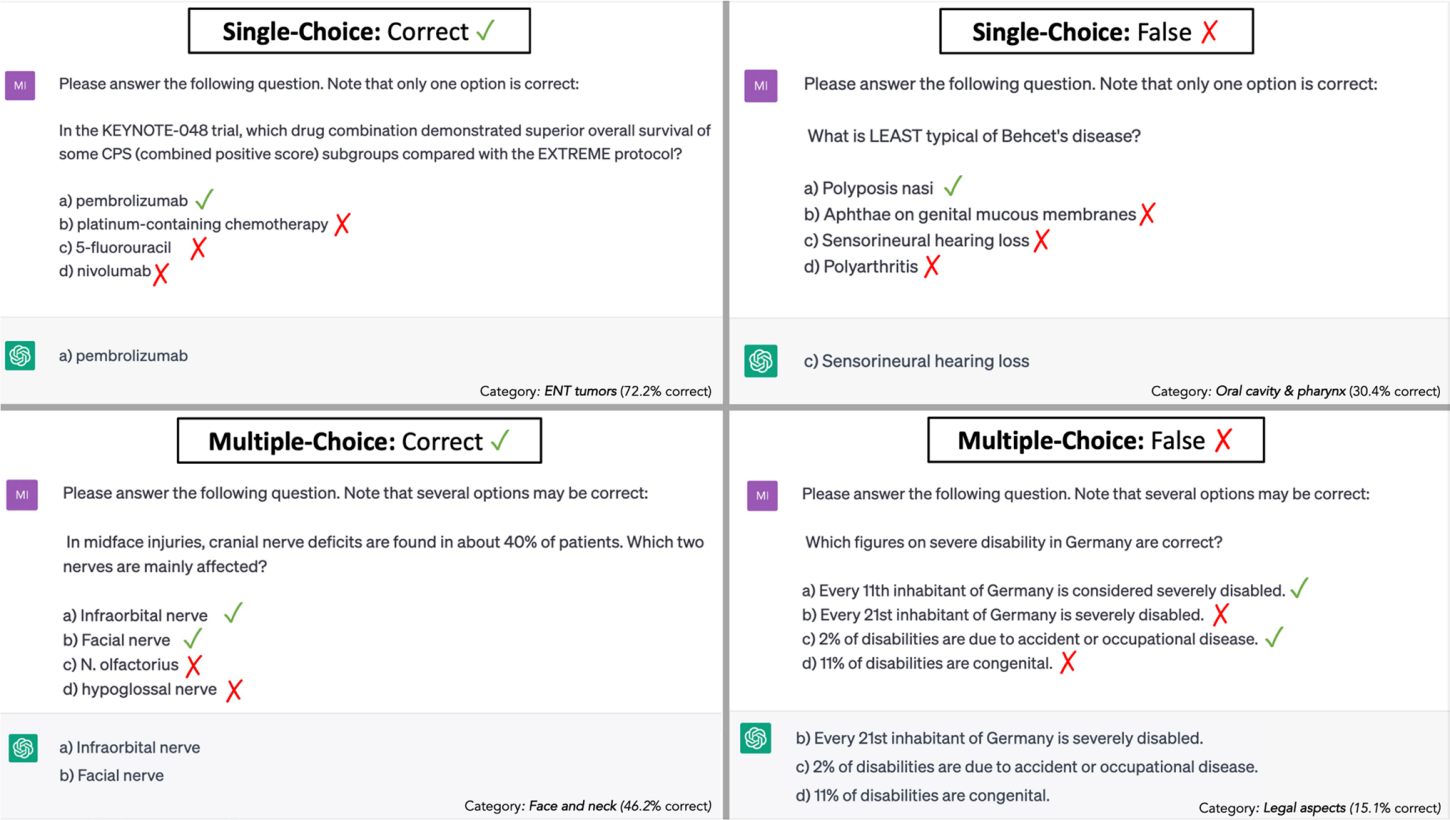
Examples of ChatGPT prompts for both multiple-choice and single-choice style questions, with correct and false responses indicated for each type of question

Subsequently, the responses generated by ChatGPT were evaluated to determine their accuracy, i.e., whether they matched the answers provided by the online study platform. For multiple-choice style questions, a response was considered correct only if all four options were accurately identified as either correct or false (Figs. 1 and 2). The collected data were then compiled into a dedicated datasheet for further statistical analyses.

### Statistical analysis

Differences between question style and categories were determined using Pearson’s chi-square test. The statistical analysis was conducted with SPSS Statistics 25 (IBM, Armonk, NY, USA) and a two-tailed *p* value of ≤ 0.05 was deemed to indicate statistical significance.

## Results

### Rate of correct and incorrect answers

Of the 2576 questions submitted to ChatGPT, 1475 questions (57%) were answered correctly, and 1101 questions (43%) were answered incorrectly, regardless of question style or category.

### Question style

ChatGPT answered a total of 2097 single-choice style questions, of which 1313 questions (63%) were answered correctly, and 784 questions (37%) were answered incorrectly. By contrast, out of the 479 multiple-choice style questions, ChatGPT answered 162 questions (34%) correctly and 317 questions (66%) falsely. A statistically significant difference (*p* < 0.001) was noted between both question styles.

### Question category

**Fig. 3:**
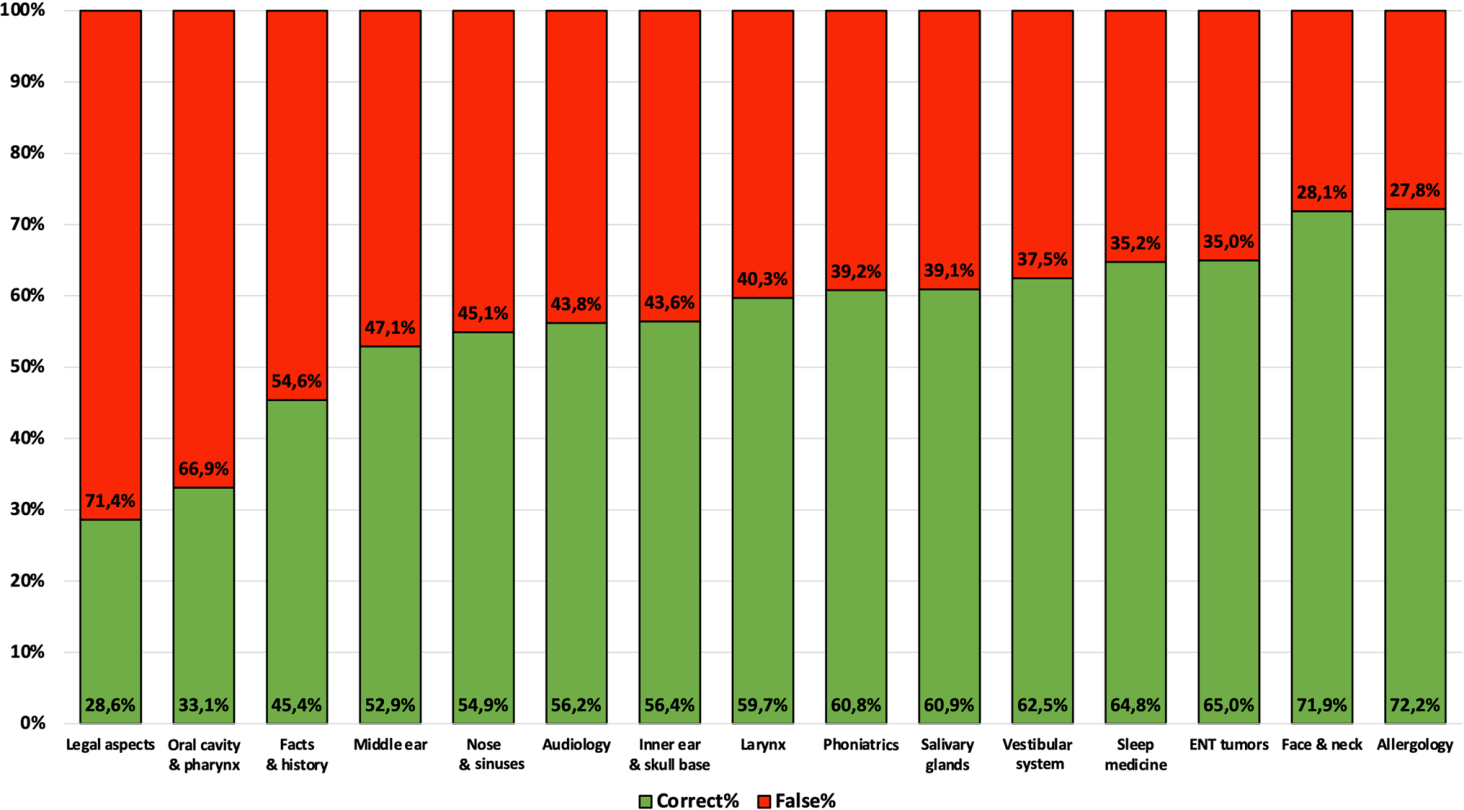
Stacked bar graphs displaying the correct and false response rates for each otolaryngology subspecialty. The correct response rates are represented by green bars, while the false response rates are represented by red bars. The subspecialties are ordered in ascending order based on their correct response rates

**Fig. 4:**
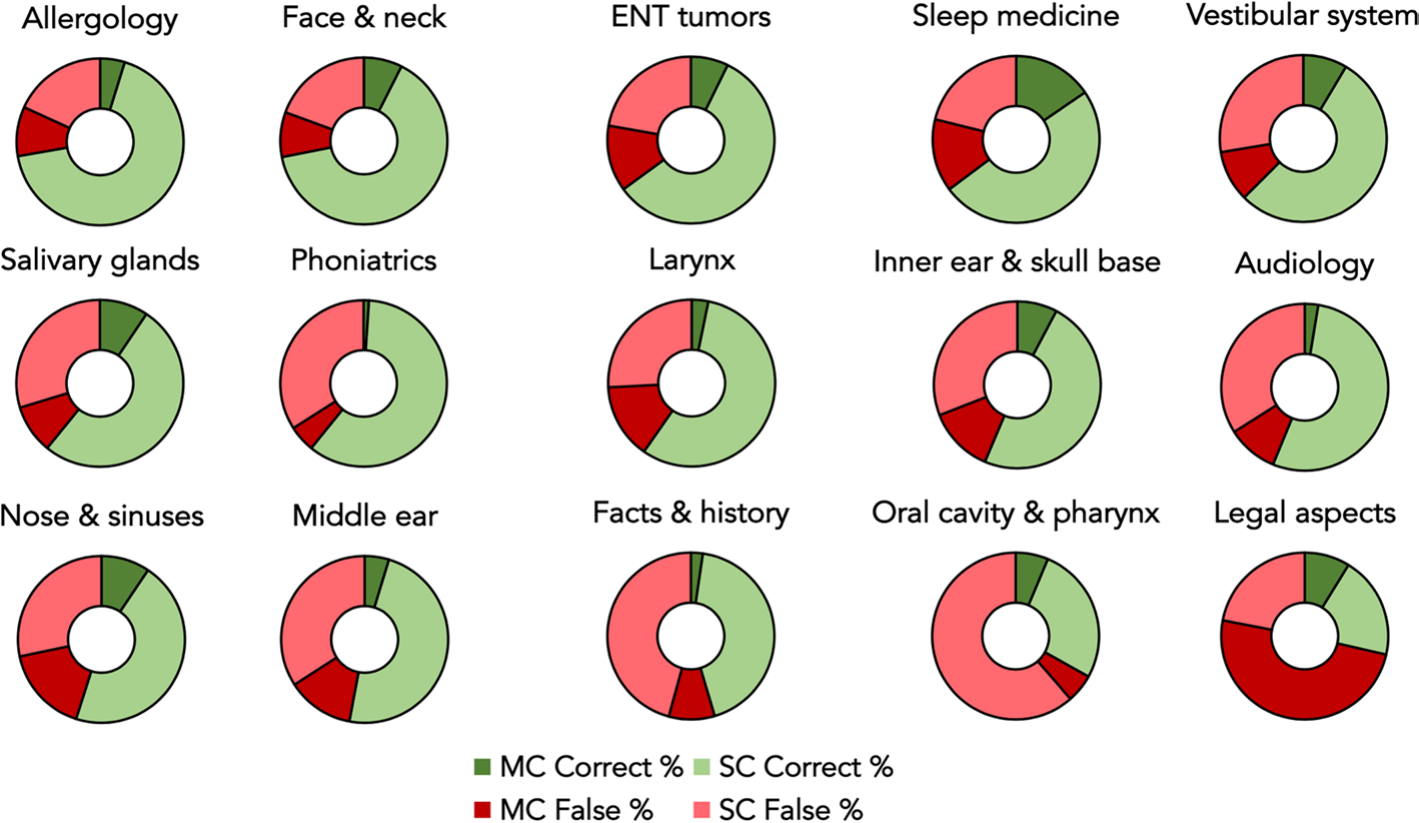
Donut charts illustrating the correct versus false rates for multiple-choice and single-choice questions, stratified by otolaryngology subspecialty. The correct rates are represented by the green sections of the charts, while the false rates are represented by the red sections. The size of each donut chart is proportional to the total number of questions in each otolaryngology subspecialty

When investigating question categories for the different otolaryngology subspecialties, the correct versus incorrect response rates, listed in descending order, were as follows: allergology (*n* = 151; 72% correct vs. *n* = 58; 28% false), face and neck (*n* = 174; 72% correct vs. *n* = 68; 28% false), ENT tumors (*n* = 152; 65% correct vs. *n* = 82; 35% false), sleep medicine (*n* = 46; 65% correct vs. *n* = 25; 35% false), vestibular system (*n* = 95; 63% correct vs. *n* = 57; 38% false), salivary glands (*n* = 84; 61% correct vs. *n* = 54; 39% false), phoniatrics (*n* = 59; 61% correct vs. *n* = 38; 39% false), larynx (*n* = 74; 60% correct vs. *n* = 50; 40% false), inner ear and skull base (*n* = 124; 56% correct vs. *n* = 96; 44% false), audiology (*n* = 86; 56% correct vs. *n* = 67; 44% false), nose and sinuses (*n* = 134; 55% correct vs. *n* = 110; 45% false), middle ear (*n* = 90; 53% correct vs. *n* = 80; 47% false), oral cavity and pharynx (*n* = 42; 33% correct vs. *n* = 85; 67% false), and legal aspects (*n* = 26; 29% correct vs. *n* = 26; 71% false), with a *p* value of < 0.001 indicating statistically significant differences between the categories (Figs. 3 and 4). Table 1 presents the results stratified by question category and style.

**Table 1:** Study results for each question category, stratified by question style (multiple choice vs single choice)

Categories	Total	Correct (%)	False (%)	MC	Correct (%)	False (%)	SC	Correct (%)	False (%)	*p* value
Middle ear	170	52.90	47.10	30	26.70	73.30	140	58.60	41.40	0.001*
Oral cavity & pharynx	127	33.10	66.90	15	53.30	46.70	112	30.40	69.60	0.76
Nose & sinuses	244	54.90	45.10	64	35.90	64.10	180	61.70	38.30	< 0.001*
Salivary glands	138	60.90	39.10	26	50.00	50.00	112	63.40	36.60	0.207
Allergology	209	72.20	27.80	30	33.30	66.70	179	78.80	21.20	< 0.001*
Larynx	124	59.70	40.30	22	18.20	81.80	102	68.60	31.40	< 0.001*
Facts & history	304	45.40	54.60	34	20.60	79.40	270	48.50	51.50	0.002*
Face & neck	242	71.90	28.10	39	46.20	53.80	203	76.80	23.20	< 0.001*
ENT tumors	234	65.00	35.00	47	36.20	63.80	187	72.20	27.80	< 0.001*
Inner ear & skull base	220	56.40	43.60	45	37.80	62.20	175	61.10	38.90	0.005*
Legal aspects	91	28.60	71.40	53	15.10	84.90	38	47.40	52.60	0.001*
Vestibular system	152	62.50	37.50	28	46.40	53.60	124	66.10	33.90	0.052
Sleep medicine	71	64.80	35.20	21	52.40	47.60	50	70.00	30.00	0.156
Audiology	153	56.20	43.80	19	21.10	78.90	134	61.20	38.80	0.001*
Phoniatrics	97	60.80	39.20	6	16.70	83.30	91	63.70	36.30	0.022*
Total	2576	57.30	42.70	479	33.80	66.20	2097	62.60	37.40	

Statistically significant differences between question styles are indicated by asterisks (*) and calculated using Pearson’s chi-square test

*MC* multiple-choice, *SC* single-choice

## Discussion

Language-based AI models, such as ChatGPT, are of increasing popularity due to their ability to maintain context and engage in coherent conversations. ChatGPT has been trained using deep learning techniques and a large amount of text data from online sources up until September 2021. Notably, its performance continues to improve through ongoing user interaction and reinforcement learning. In this study, we demonstrated the applicability of ChatGPT in the field of otolaryngology by evaluating its performance in answering quiz-style questions specifically designed for the German otolaryngology board certification examination.

Prior to the public release of ChatGPT, several studies analyzed the potential of AI models in answering medical licensing exam questions. For example, Jin et al. noted an accuracy rate of only 37% when evaluating a dataset comprising 12,723 questions from Chinese medical licensing exams [12]. Ha et al. reported a lower accuracy rate of 29% based on their analysis of 454 questions from the United States Medical Licensing Exams (USMLE) Step 1 and Step 2 exams in 2019 [13].

Reaching beyond the boundaries of one-dimensional question-answering tasks, ChatGPT pushed the traditional boundaries of one-dimensional question-answering tasks and, therefore, represents a significant leap forward in web-based remote knowledge access with broad practicality for both medical laymen and experts. Gilson et al. demonstrated that ChatGPT performs comparably or even surpasses previous models when confronted with questions of similar difficulty and content [14]. These findings highlight the improved ability of the model to generate accurate responses through integrative thinking and medical reasoning. Accordingly, a recent study evaluating ChatGPT’s performance across all three USMLE steps (namely, Step 1, Step 2CK, and Step 3) revealed a substantial level of agreement and provided valuable insights through the comprehensive explanations generated by ChatGPT [15]. It is worth noting that the authors addressed bias concerns by clearing the AI session prior to presenting each question variant and requesting forced justification only as the final input.

A major strength of our study lies in the extensive dataset of 2576 quiz questions, including both single-choice and multiple-choice formats, across 15 distinct otolaryngology subspecialties. These questions, initially designed for the German board certification examination, are characterized by a higher level of difficulty compared to typical otolaryngology questions in medical licensure examinations.

Despite the complex nature of the questions, ChatGPT was able to answer more than half of all questions correctly. Of note, specifically in single-choice questions, ChatGPT was most successful, with over 60% rate of correct answers. In contrast, multiple-choice questions appeared to be a greater hurdle for ChatGPT: only one third of this question type could be answered correctly. This finding of a significant difference in performance between question formats is consistent with results reported by Huh, who highlighted ChatGPT’s inherent difficulty in accurately answering multiple-choice questions [16]. These observed disparities in the correctness of ChatGPT’s responses when it comes to single-choice and multiple-choice questions may be attributed to the underlying operational principles of ChatGPT’s technology. One may, therefore, hypothesize that ChatGPT is designed to analyze the available options and prioritize the most plausible correct answer, rather than independently evaluating the validity of each answer option.

In addition, our analysis included an examination of ChatGPT’s performance across diverse otolaryngology subspecialties, revealing marked variations in the rates of correct responses. For instance, ChatGPT yielded the highest rate of correct answers in the field of allergology, whereas less than 3 in 10 questions regarding legal aspects were answered correctly by ChatGPT. These significant disparities in performance across subspecialties could be attributed to the varying availability and quality of training data for each category. It is important to consider that the question category “legal aspects”, which referred to German medical law, presented a challenge for ChatGPT due to its reliance on a potentially more limited literature database. In contrast, otolaryngology subspecialties with greater rates of correct ChatGPT responses may have benefited from more extensive data sources and a broader pool of retrievable information. Moreover, categories associated with high correct/false response ratios, such as allergology, are likely to be topics for which ChatGPT users frequently seek medical advice. This underscores the potential for continuous improvement through regular user interaction, thereby broadening the model’s armamentarium while sharpening its accuracy.

In a recent study investigating the response accuracy of otolaryngology residents utilizing the same database but incorporating image-based questions, the results revealed a 65% correct answer rate [17]. Similar to our findings, the allergology category emerged as one of the top-performing categories, with nearly 7 in 10 questions being answered correctly by the residents. However, consistent with our study, the nose and sinuses category and the facts and history category proved to be more challenging. These findings suggest that while AI has made considerable advancements, it still falls short of matching the capabilities of its human counterparts.

As an educational resource, the performance of ChatGPT indicated potential efficacy in offering educational assistance in specific subspecialties and question formats. Nevertheless, the study also underscored aspects that need improvement. Notably, ChatGPT delivered a considerable number of incorrect responses within specific otolaryngology subdomains, rendering it unreliable as the sole resource for residents preparing for otolaryngology board examination.

In addition to the complexity of its usage, concerns have been raised about the potential misuse of AI tools like ChatGPT to cheat or gain unfair advantages during medical examination tests. It is important to clarify that our study aimed to evaluate the effectiveness of ChatGPT as a tool for test preparation, not to encourage its use during the actual examination process.

Our results revealed that, given its limitations and inconsistent performance across different subspecialties and question formats, ChatGPT does not currently provide a significant unfair advantage to test-takers. This conclusion, however, might not remain static as AI models like ChatGPT continue to evolve. The progression of these models, driven by improved training data and increasingly sophisticated algorithms, heralds the arrival of more accurate language models capable of generating contextually relevant responses. This development, in turn, presents fresh ethical dilemmas regarding their application in educational settings.

Despite these challenges, the key takeaway is the importance of integrating ChatGPT into a wider learning strategy. This approach should supplement AI-based learning with traditional educational methods such as textbooks, lectures, and one-on-one sessions with subject matter experts. This combination ultimately ensures a well-rounded learning experience, while also mitigating potential reliability and ethical issues associated with the sole use of AI tools for educational purposes.

### Limitations

When interpreting the results and drawing conclusions, the study’s inherent limitations must be considered. The use of a single online learning platform that incorporates a mononational question database exclusively focused on a specific subfield of medicine limits the generalizability and transferability of our results to other medical disciplines. In addition, the absence of implementing the session clearing process before each question in our study has the potential to significantly impact the accuracy of the responses provided by ChatGPT, as this process aims to remove biases or influences from prior questions. Future investigations are needed to explore potential improvements in the rates of correctly answered questions by employing a well-defined question database within a longitudinal study design. Such an approach would offer valuable insights into ChatGPT’s capacity to learn and improve over time through continuous user interaction.

## Conclusion

The study’s findings underscore the potential of AI language models like ChatGPT as a supplemental educational tool for otolaryngology knowledge mining and board certification preparation. However, the study also identified areas for improvement, as ChatGPT provided false answers to a substantial proportion of questions in specific otolaryngology subdomains. This highlights the need for further refinement and validation of the model. Future research should focus on addressing the limitations identified in our study to improve the efficacy of ChatGPT as an educational tool in broader educational contexts. The integration of AI language models should be approached with caution and in close cooperation with human experts to ensure their reliability and accuracy.

## Data Availability

The data presented in this study are available on request from the corresponding author. The data are not publicly available due to reasons of legal data protection.
